# Synergetic Effect of Potassium, Biochar and Cattle Manure on the Growth and Yield of Maize, and Soil Physio-Chemical Characteristics

**DOI:** 10.3390/plants13233345

**Published:** 2024-11-28

**Authors:** Zeqiang Shao, Xiuzhi Zhang, Jamal Nasar, Harun Gitari

**Affiliations:** 1 Jilin Institute of Chemical Technology, College of Resource and Environment Engineering, Jilin City 132022, China; zeqiangshao@126.com; 2 Institute of Agricultural Resources and Environment, Jilin Academy of Agricultural Sciences (Northeast Agricultural Research Center of China), Changchun 130033, China; 3 Institute of Rice Industry Technology Research, College of Agriculture, Guizhou University, Guiyang 550025, China; jamalnasar554@gmail.com; 4 Department of Agricultural Sciences and Technology, Kenyatta University, Nairobi 00100, Kenya; hgitari@gmail.com

**Keywords:** biochar, cattle manure, crop yield, soil fertility, potassium dynamics

## Abstract

Biochar (BC) and cattle manure (CM) are carbon-nutrient-rich organic substances and have long been used to improve crop yield and soil fertility. Nevertheless, their combined effect with potassium (K) fertilizer remains unknown. Against the previous context, a 2-year (2021–2022) field experiment was conducted to assess the effect of K fertilization coupled with BC and CM on the growth and yield of maize and soil physio-chemical characteristics. The K application combined with BC and CM increased (*p* ≤ 0.05) the majority of the growth indices of maize crop compared with CK. Compared with CK, the combined application of K (60 kg K ha^−1^) with BC and CM resulted in an increased number of seeds cob^−1^ by up to 451 and 465, and up to 383 and 396, the 1000-seed weight up to 22 and 23 g, and up to 27 and 34 g, and the grain yield up to 1979 and 2900 and up to 3240 and 3341 kg ha^−1^, respectively, in 2021 and 2022. The integrated application of these inputs increased the chlorophyll of maize crops by 29 and 36% and by 30 and 44%, respectively, in 2021 and 2022. Such application also increased the photosynthetic activities of maize such as transpiration rate (Tr), stomatal conductance (Gs), and photosynthetic rate (Pn) by 21 and 23%, 143 and 110%, and by 64 and 66% in 2021 and by 19 and 30%, 163 and 118%, and by 63 and 72% in 2022. Similarly, the combined application of K, BC, and CM increased the K uptake of maize due to an increase in the soil extractable K. Equally, soil total N and organic matter improved under the combined application of K, BC, and CM. However, it did not affect the soil extractable P in 2021 but increased it in 2022. Conversely, these applications reduced (*p* < 0.05) the soil electrical conductivity, sodium adsorption ratio, and bulk density. This suggests that K fertilization combined with BC and CM enhances the growth and yield of maize by improving the soil nutrients availability, increasing soil organic matter, and enhancing soil structure and moisture retention.

## 1. Introduction

Maize (*Zea mays* L.) is the major cereal crop grown for food and forage production worldwide [1,2]. The demand for the crop has recently increased due to the rapid growth of the livestock industry [3]. Although maize production has risen by 460% from 1961 to 2019, there is still a need to increase its production further by 40% by 2050 [4,5]. Therefore, to warrant a supply of high-quality forage and cereal, and gratify the ever-increasing demand for meat products, an increase in maize production is inevitable.

Potassium (K) is a primary macronutrient that plays an imperative role in plant growth and development [6]. It is involved in plant photosynthesis and enzyme activity, besides protecting the plants from nematodes and disease attacks [7]. Plants that lack K show severe physiological disorders, which drastically affect plant growth and yield [8]. A sufficient application of K upsurges the growth and yield of crops by improving their nutrient and water uptake, and their use efficiencies [7]. In addition, K fertilization helps recover the plant’s physio-morphological performance under drought and aids in the transportation of sugar and starch, in addition to being vital in cellulose formation, maintaining the plant’s turgor, increasing the protein content of plants, root growth, and reducing water loss [6,9]. For example, K fertilization at 120 kg ha^−1^ has been shown to increase significantly the growth and yield indices of wheat crops such as height by up to 97 cm, maximum quantum yield of photosystem II (Fv/Fm) (0.83), plant performance index (PI) (5.49), chlorophyll SPAD values (58.63), membrane stability index (MSI) (34.57), seed yield (5.04 t ha^−1^), straw yield (9.04 t ha^−1^), and water productivity (0.99) under salinity stress in the semi-arid condition of Egypt [10]. It also increased the uptake of nutrients (i.e., N, P, K, Ca, Mg, Fe, Mn, and Zn) in wheat crops [10]. Several other studies have shown that K fertilization at optimal dosage can help improve the growth and yield of crops [10,11,12].

Cattle manure (CM) is the waste material produced by cattle, primarily consisting of feces and urine, along with bedding materials like straw or sawdust [13]. It is a valuable organic fertilizer used in agriculture, and useful in providing essential nutrients to the soil and improving its overall health [13]. It works as a soil conditioner, by increasing soil organic carbon (C), and nutrient content besides enhancing soil functioning capacity [14]. Cow dung, the excrement produced by cows has been one of the most used cattle manures in agriculture for several decades. It ought to be properly processed and recycled through land application to fulfil crop nutrient demands while reducing mineral fertilizer requirements [15]. Integrating such CM into soil results in sustainable soil health through the recycling of the plants’ macro and micronutrients [13]. Such a practice results in better crop growth and development, thereby increasing crop yields [16,17]. More so, CM enhances soil physical characteristics by increasing macropore volume, aggregate stability, and saturated hydraulic conductivity [18]. In addition, CM, in integration with optimal mineral fertilizers, can help to increase crop yields and reduce greenhouse gas emissions to a certain extent [19]. For instance, CM (i.e., cow dung at 9 t ha^−1^) together with mineral fertilizers (i.e., N, P, and K) has been shown to increase the growth and yield of maize crops mainly by improving the plants’ uptake of nutrients such as N, P, and K, and by increasing the available soil nutrients [13]. Some studies have suggested that CM is a better soil conditioner, which could help improve the overall functionality of the soil, and as a result, plants grown on the same soil could have better growth and yield [19,20,21].

Biochar (BC), is a carbon-rich substance produced from a wide range of natural waste resources, including forest, agricultural, industrial by-products, and municipal solid waste, by pyrolysis under low oxygen environments [22,23]. As a low-cost technology product, BC application in soil upsurges farm economic returns by boosting crop productivity [24,25]. Moreover, the application of BC in soil improves soil aeration and water retention, creating a better environment for plant root development, and its porous structure allows for improved drainage and root penetration [26]. The high cation exchange capacity (CEC) of BC helps retain the essential nutrients in soil, reduces nutrients leaching, and make them more available to plants, thereby increasing crop yield. Likewise, BC modify the majority of the soil physio-chemical characteristics, changes soil enzymatic activities, and microbial community structures, thereby improving the soil quality and health [27,28,29]. The application of BC at 20 t ha^−1^ has been reported to improve maize yield and water relation traits under drought conditions [30]. Its integration with compost has also been shown to improve the growth and yield of wheat crops [31]. In addition, BC in combination with inorganic nitrogen fertilizer has drastically increased the chlorophyll content, chlorophyll fluorescence, and photosynthetic activities of rice crops [32,33]. Several other studies have shown that BC, either alone or in combination with inorganic fertilizers, can significantly improve the growth and yield of crops and soil fertility status [34,35,36].

Northeast China is an important commodity base of agriculture and animal husbandry [2]. Maize is a major cereal crop grown in this area for food and forage production [37]. However, the increased use of chemical fertilizers, especially ammonium-based ones, has detrimental effects on the environment and agricultural ecosystem, including on soil acidification [38]. Therefore, to increase maize yield, and improve soil fertility and farmland ecosystem while protecting the environment, it is imperative to opt for agronomic techniques that have less harm to the agricultural ecosystem and environment. Considering the potential of organic amendments such as CM and BC in agricultural production and soil quality improvement, their combination with the optimal K fertilizer could be a better option for improving maize growth and yield, and the soil fertility status. The effect of K, BC, and CM alone, or the combination of any two, is well investigated in agriculture, plants, and soil health [7,31,39]. Nonetheless, in the literature, there is no evidence of the effect of K fertilizers combined with BC and CM on maize growth and yield and soil physio-chemical characteristics. Hence, the current study was designed to examine the effects of K fertilizers combined with BC and CM on maize growth and yield and some soil physio-chemical characteristics. We hypothesized that K fertilizer, combined with BC and CM, improves maize growth and yield by improving the soil quality parameters. The main objective of this research was to study the effect of BC and CM, along with K fertilizer, on the physical agronomic indices of maize, K uptake, and some soil physio-chemical parameters.

## 2. Results

### 2.1. Growth Indices

**Figure 1:**
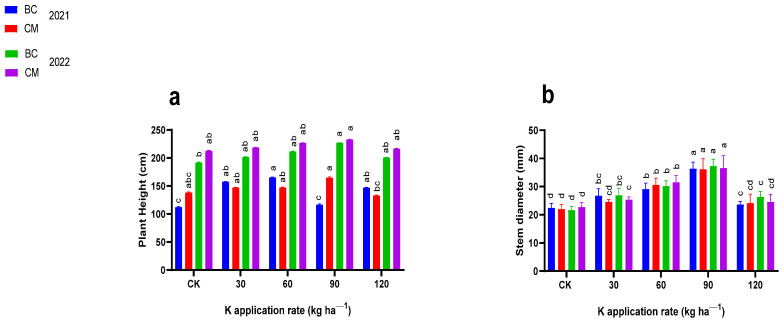
Plant height and stem diameter of maize crops under potassium (K) fertilization combined with biochar and cattle manure in the years (**a**) 2021 and (**b**) 2022. BC; biochar, CM; cattle manure, CK; control treatment. Dissimilar lowercase letters on the graph represent the differences among the means at LSD test *p* ≤ 0.05.

**Figure 2:**
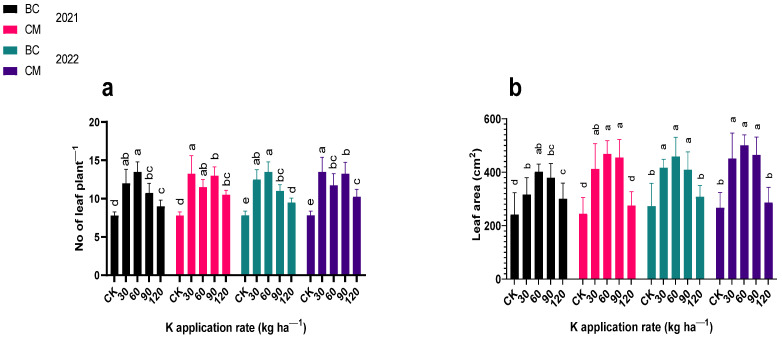
Number of leaves and leaf area of maize crops under potassium (K) fertilization combined with biochar and cattle manure in the years (**a**) 2021 and (**b**) 2022. BC; biochar, CM; cattle manure, CK; control treatment. Dissimilar lowercase letters on the graph represent the differences among the means at LSD test *p* ≤ 0.05.

The K fertilization combined with BC and CM influenced (*p* ≤ 0.05) the growth indices, such as stem diameter (mm), plant height (cm), the number of leaves, and leaf area (cm^−2^) of the maize crop (Figure 1 and Figure 2). When compared to CK, the maize plants treated with 90 kg K ha^−1^ combined with CM were the tallest (165.27 cm), and with 60 kg K ha^−1^ combined with BC, emerged as the second tallest (164.93 cm) in 2021. In 2022, the maize height increased to 233.25 cm and 227.34 cm when treated with 90 kg K ha^−1^ combined with CM and BC, respectively, as compared to CK. The maize stem diameter increased by 64 and 62% and by 83 and 69% in the plants treated with 90 kg K ha^−1^ combined with CM and BC, respectively, in 2021 and 2022 as compared to CK. Maize plants had more leaves (i.e., 13.5, 13.25, and 13.5) in plants treated with 60 kg K ha^−1^ combined with BC, and 30 kg K ha^−1^ combined with CM, respectively, in 2021 and 2022 than maize plants in the CK. When compared to CK, the leaf area of maize crops increased by 66 and 92% in the plants treated with 60 kg K ha^−1^ combined with BC and CM respectively in 2021 and increased by 68 and 88% in the plants treated with 30 kg K ha^−1^ combined with BC and CM, respectively, in 2022.

### 2.2. Yield and Yield Indices

**Figure 3:**
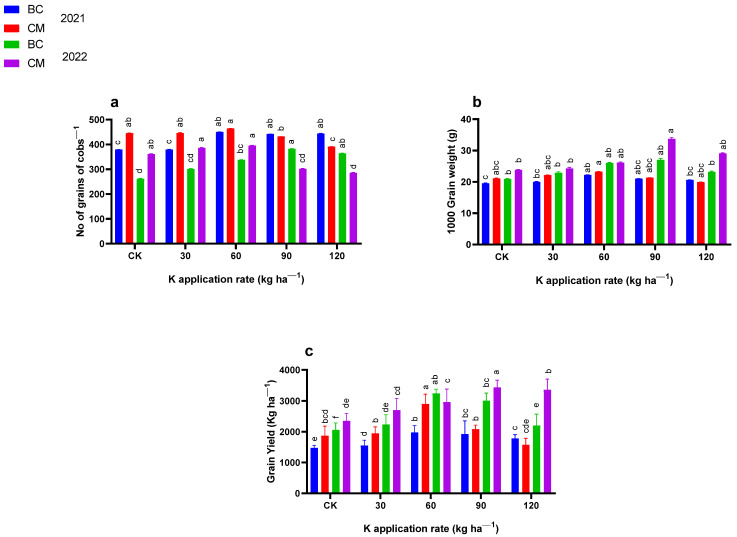
(**a**) Number of cobs^−1^, (**b**) 1000-grain weight (**c**) and grain yield of maize under the application of potassium (K) fertilization combined with biochar and cattle manure in the years 2021 and 2022. BC; biochar, CM; cattle manure, CK; control treatment. Dissimilar lowercase letters on the graph represent the differences among the means at LSD test *p* ≤ 0.05.

The K fertilizer combined with BC and CM affected (*p* ≤ 0.05) yield indices such as 1000-grain weight (g), the number of grains per cob^−1^, and grain yield (kg ha^−1^) of maize crop (Figure 3). Compared to CK, the maize plant treated with 60 kg K ha^−1^ combined with BC and CM produced the maximum number of grains per cob^−1^ (451 and 464.5) in 2021. However, this number increased to 382.25 in the plants that had received 90 kg K ha^−1^ combined with BC, and up to 395.5 in the plants treated with 60 kg K ha^−1^ combined with CM in 2022 as compared to CK. Furthermore, the highest 1000-grain weight 22.2 and 23.2 g was recorded in the plants that had received 60 kg K ha^−1^ combined with BC and CM in 2021, respectively, as compared to CK. However, in 2022, the 1000-grain weight (g) of the maize crop increased up to 27 and 33.7 g in the plants that had received 90 kg K ha^−1^ combined application of BC and CM, respectively, as compared to CK.

Compared to CK, the grain yield of maize crops increased up to 1978.5 and 2900.3 kg ha^−1^ when treated with 60 kg K ha^−1^ combined with BC and CM in 2021, respectively. However, the grain yield of the maize crop increased up to 3240.4 kg ha^−1^ under the combined application of 60 kg K ha^−1^ with BC, and up to 3340.7 kg ha^−1^ under the combined application of 90 kg K ha^−1^ with CM in 2022 as compared to CK.

### 2.3. Chlorophyll SPAD Values and Photosynthetic Activities

**Figure 4:**
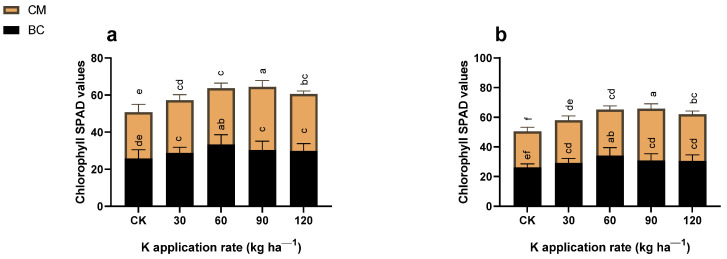
Chlorophyll SPAD values of maize under potassium (K) fertilization combined with biochar and cattle manure in the years (**a**) 2021 and (**b**) 2022. BC; Biochar, CM; cattle manure, CK; control treatment. Dissimilar lowercase letters on the graph represent the differences among the means at LSD test *p* ≤ 0.05.

**Figure 5:**
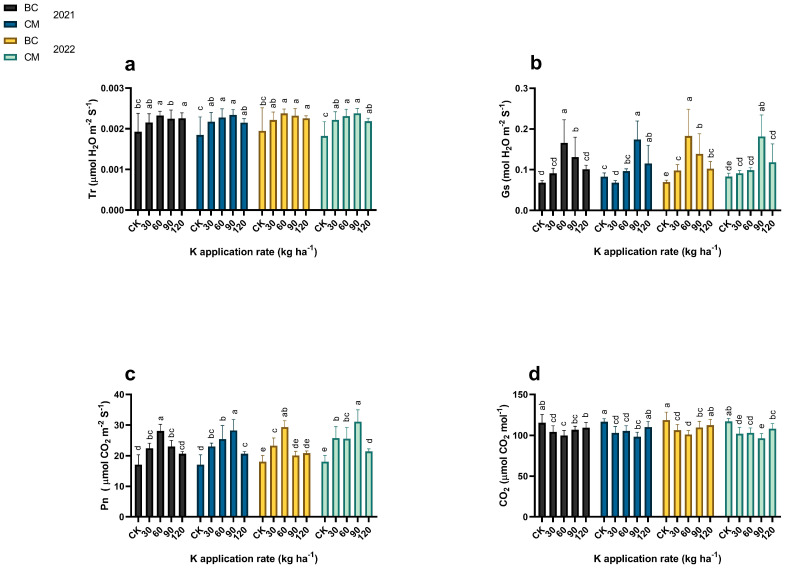
Photosynthetic activities of maize under potassium (K) fertilization combined with biochar and cattle manure in the years 2021 and 2022. (**a**) Tr: transpiration rate, (**b**) Gs; stomatal conductance, (**c**) Pn; photosynthetic rate, (**d**) CO_2_; intercellular carbon dioxide. BC; Biochar, CM; cattle manure, CK; control treatment. Dissimilar lowercase letters on the graph represent the differences among the means at LSD test *p* ≤ 0.05.

The K fertilizer, combined with BC and CM, affected (*p* ≤ 0.05) the chlorophyll SPAD values and the photosynthetic activities of the maize crop in the years 2021 and 2022 (Figure 4 and Figure 5). When compared to CK, the chlorophyll SPAD values of maize crops increased by 29% under 60 kg K ha^−1^ coupled with BC, and by 36% under 90 kg K ha^−1^ combined with CM in 2021. However, these values further increased by 30 and 44% under the same treated plant in 2022 as compared to CK. Similarly, the photosynthetic activities, such as Tr, Gs, and Pn, increased by 21 and 23%, 143 and 110%, and 64 and 66% under 60 kg K ha^−1^ coupled with BC and 90 kg K ha^−1^ combined with CM, respectively, in 2021 as compared to CK. However, these values increased by 19 and 30%, 163 and 118%, and by 63 and 72% under the treatment plots respectively in 2022 as compared to CK. In contrast, K fertilization combined with BC and CM reduced the CO_2_ by 13 and 16%, and by 15 and 17% under 60 kg K ha^−1^ combined with BC and 90 kg K ha^−1^ combined with CM respectively in 2021 and 2022, in contrast with CK.

### 2.4. Total K Uptake

**Figure 6:**
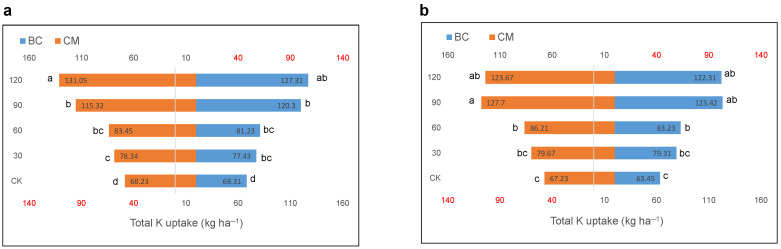
Total potassium (K) uptake by maize crop under the combined application of biochar and cattle manure along with potassium fertilizer in the years (**a**) 2021 and (**b**) 2022. BC; Biochar, CM; cattle manure, CK; control treatment. Dissimilar lowercase letters on the graph represent the differences among the means at LSD test *p* ≤ 0.05.

The K fertilization combined with BC and CM had an impact (*p* ≤ 0.05) on the total K uptake of maize crops in both years. The application of K combined with BC and CM increased the total K uptake of the maize crop (Figure 6). The maximum total K uptake of 131.05 and 127.31 kg ha^−1^ was noted in plants that had received 120 kg K ha^−1^ combined with CM and BC, respectively, in 2021 as compared to CK. However, the respective values in 2022 were 127.70 and 123.42 kg ha^−1^ in the plants that had received 90 kg K ha^−1^ combined with CM and BC, respectively, as compared to CK.

### 2.5. Soil Characteristics

The K fertilization combined with BC and CM influenced (*p* ≤ 0.05) the majority of the soil parameters in both years (Table 1). The results showed that the application of K fertilizer combined with BC and CM did not significantly affect the soil pH. However, the application of K at the rate of 90 kg K ha^−1^ combined with BC reduced the soil EC from 660.0 to 421.20 μscm^−1^, and from 321.3 to 206.6 μscm^−1^ under the 60 kg K ha^−1^ combined with CM in 2021 as compared to CK. In 2022, the soil EC decreased to 613.1 μscm^−1^ under the 60 kg K ha^−1^ combined with CM, and to 621.00 μscm^−1^ under the 90 kg K ha^−1^ combined with BC as compared to CK. Moreover, the K application at 120 kg K ha^−1^ combined with BC and CM reduced the SAR to 2.58 and 1.40, respectively in 2021 as compared to CK. However, in 2022, the SAR decreased to 1.68 in the plot that had received 30 kg K ha^−1^ combined with BC and reduced to 0.89 in the plot that had received 90 kg K ha^−1^ combined with CM as compared to CK. Furthermore, the maximum soil bulk density (BD) of 1.249 g cm^−3^ was reported in the plot that had received 90 kg K ha^−1^ combined with CM, and the soil BD of 1.245 g cm^−3^ was noticed in the plot treated with 60 kg K ha^−1^ combined with BC in 2021 as compared to CK. In the year 2022, the maximum soil BD of 1.410 g cm^−3^ was noticed in the plots that had received 90 kg K ha^−1^ combined with CM, and the soil BD of 1.430 g cm^−3^ was noticed in the plots that had received 60 kg K ha^−1^ combined with BC as compared to CK. In addition, the maximum organic matter of 0.62 and 0.91% was recorded in the plot treated with 30 kg K ha^−1^ combined with BC and 120 kg K ha^−1^ combined with CM respectively in 2021 as compared to CK. However, in 2022, the maximum organic matter of 0.78% and 0.98% was recorded in the plot treated with 90 kg K ha^−1^ combined with BC and 120 kg K ha^−1^, respectively, as compared to CK.

### 2.6. Soil Available Nutrients 

The K fertilization combined with BC and CM influenced (*p* ≤ 0.05) the soil available nutrients in both years (Table 2). The K application at 120 kg K ha^−1^ combined with CM and BC increased the soil total nitrogen up to 0.045 and 0.027%, respectively in 2021 as compared to CK. However, the soil total nitrogen increased up to 0.049% in plots that had received 120 kg K ha^−1^ combined with CM and increased up to 0.039% in plots that had received 90 kg K ha^−1^ combined with BC as compared to CK. Conversely, the K fertilization combined with BC and CM did not affect the soil extractable P in the year 2021 but had an impact (*p* ≤ 0.05) in 2021. When compared to CK plots, the highest soil extractable P of 8.11 mg kg^−1^ was noted in the plots that had received 90 kg K ha^−1^ combined with BC, and the second highest soil extractable P of 7.97 mg kg^−1^ was reported in the plots that had received 60 kg K ha^−1^ combined with CM.

Moreover, the maximum K retention in soil 209.07 mg kg^−1^ was found in the soil treated with 90 kg K ha^−1^ combined with CM, and 200.09 mg kg^−1^ was noticed in the plots treated with 120 kg K ha^−1^ combined with BC in 2021 as compared to CK. However, these values were 221.35 and 213 mg kg^−1^ in the same treated plots, respectively, in 2022 as compared to CK.

### 2.7. Correlation of Different Factors with K

**Figure 7:**
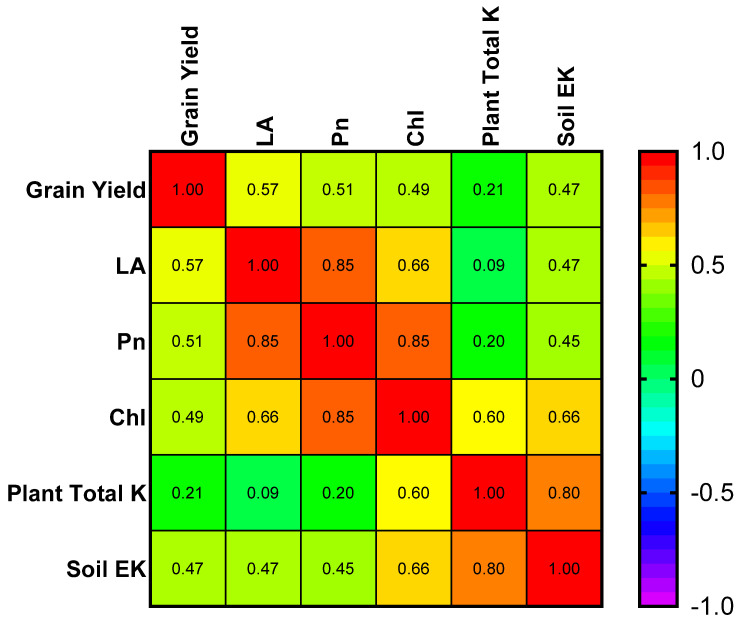
Correlation matrix of maize grain yield with leaf area (LA), photosynthetic rate (Pn), chlorophyll (Chl), plant total potassium uptake (K), and soil extractable potassium (soil EK).

The correlation analysis was used to determine the relationship of maize grain yield with leaf area, photosynthetic rate, chlorophyll, total K uptake of maize, and soil extractable K (Figure 7). The correlation matric results showed that maize grain yield is positively correlated with leaf area, photosynthetic rate, chlorophyll, total K uptake of maize, and soil extractable K.

## 3. Discussion

### 3.1. Growth and Yield Indices of Maize

Potassium is a major macronutrient, which is essential for plant growth and development [7]. BC and CM are naturally available carbon-nutrient-rich substances that are used as soil amendments to improve soil fertility for better crop growth and yield production [15,22]. Thus, K in combination with BC and CM further surges the crop growth and development, and thereby the crop yield. The present study showed that the K fertilizer (i.e., 90 or 60 kg ha^−1^) combined with BC and CM increased the majority of the growth and yield indices of maize crops. These applications increased the maize yield by 34, 55, 57, and 46% in 2021 and 2022. This could mean that BC enhances soil aeration and water retention, providing a better environment for root development [25,40]. Its porous structure allows for improved drainage and root penetration [26]. In addition, the high cation exchange capacity (CEC) and pH balancing ability of BC helps retain essential plant nutrients in the soil, reduces nutrient leaching and creates a more favorable environment for plant nutrients uptake [26]. CM on the other hand is rich in plant essential nutrients (i.e., N, P, and K), and acts as a slow-release source of these fertilizers [41]. CM foster diverse microbial populations in soil, which aids in nutrients cycling and enhances soil fertility [42]. It also helps in soil carbon storage, improving soil water retention, thereby enhancing soil health and fertility [43]. Thus, integration of K, BC and CM into the soil might improve the soil quality, health, structure, and fertility, thereby improving plant growth and development, and ultimately crop yield [44]. Previous studies have shown that BC and CM, either alone or in combination with a mineral fertilizer (i.e., N, P, and K), have significantly improved plant growth and development and produced higher crop yields, which were mainly attributed to the improvement in soil nutrient availability and changes in the soil’s physio-chemical characteristics [7,31,45]. It has also been documented that the optimal application of K fertilizer combined with BC and CM works synergistically, enhancing maize plants’ nutrients availability and their uptake efficiency, leading to an increased 1000-grain weight and number of grains per cob. As a result, maize plants produced a higher yield [31]. It has been recounted that biochar application at the rate of 50 t ha^−1^ and cattle manure application at 20 t ha^−1^ increased the yield attributes such as the 1000-grain weight and the number of grains per cob, and ultimately yield [46,47], which confirmed the results of the current study. Several other studies have shown that BC and CM, either alone or in combination with mineral fertilizer, drastically improved the growth and yield indices of the crop, mainly due to an improvement in soil nutrient availability and uptake efficiency [13,48,49].

### 3.2. Chlorophyll and Photosynthetic Characteristics

Chlorophyll is a green pigment located in plant chloroplast that plays a crucial role in plant photosynthesis [50]. Photosynthetic activities refer to the process of converting light energy into plant chemical energy [50]. Any changes in chlorophyll can bring changes in plant photosynthesis [38]. In this study, the combined supply of K, together with BC and CM, increased the chlorophyll SPAD values and photosynthetic activities. This happened because of the combined applications of K, BC, and CM. Together, they helped in improving the leaf area of the maize crop, courtesy of a large number of leaves, which help plants to intercept more sunlight, hence resulting in enhanced chlorophyll and increased photosynthetic activities that drastically influence maize physiological traits and ultimately the yield of maize crops. This could also be due to the fact that these organic amendments boost the nitrogen availability, which could effectively increase the chlorophyll content, thereby enhancing the photosynthetic activities [26]. The proper amount of available plant nutrients in BC and CM helps in plant nutrients uptake, resulting in better chlorophyll and photosynthetic activity, thereby increasing crop yield [49,51,52]. As previously reported, CM at the rate of 95 m^3^ ha^−1^ had significantly improved such chlorophyll and photosynthetic indices of maize crops, and thereby the grain yield of maize crops, but under clay soil conditions it responds well with CM at the rate of 46 m^3^ ha^−1^ [53]. The BC application, along with potassium fertilizer, has also been shown to increase the grain yield of maize crops by 18–20% because of the changes that occur in the growth traits of maize crops [7]. The high amount of nutrients present in BC and CM triggers the chlorophyll and photosynthetic indices of crops, and as a result, plants produce a higher yield [54,55].

### 3.3. Total K Uptake and Its Mechanism

The current study revealed that BC and CM application, with or without K fertilizers, significantly increased the K uptake of maize crops. However, such K uptake was more evident with K fertilizers. This effect could be attributed to the presence of K in the BC and CM, which were released into the soil through mineralization, resulting in increased soil K. Thus, plant roots take up more K from the soil [23,56]. The concentration of cation in most plant tissues is dependent on the crop and impartially independent of the fertilization or soil, even though dry soil conditions severely reduce the mobile ions in roots, which impedes the transformation of soil nutrients into plant-available K [57]. Several studies have shown a significant increase in plant K uptake when treated with BC or CM [45,58,59]. Thus, this suggests that BC and CM with optimal K fertilizers could be potential organic soil amendments for the improvement of the available K in the soil and its uptake in plants, which in turn help plant growth and yield and soil quality improvement.

### 3.4. Soil Physio-Chemical Characteristics

The current study demonstrated that BC and CM application, with or without K fertilizer, significantly influenced the majority of the soil parameters. It was noticed that CM application with or without potassium fertilizers increased the EC of the soil. However, BC application increased the soil EC before plant sowing, but decreased it when plants were harvested. The increased trends of soil EC with BC application were due to the presence of salt contents in it [60]. Nevertheless, the decreased trends could be due to the addition of K fertilizer, where K reacts with NaCl forming KCl, which is less soluble as compared to NaCl [60]. Furthermore, such applications of BC and CM reduced the soil SAR. The SAR echoes the concentration of Na^+^ in relation to the square root of the concentrations of Ca^2+^ and Mg^2+^ in soil solution [61]. Organic amendment addition to soil increases the Ca^2+^ and Mg^2+^ in soil solution at marked concentrations, which causes a decline in the soil’s sodium adsorption ratio [8]. Previously, it has been shown that, after leaching the soil and amending with organic amendments, there was a significant reduction in SAR [62]. The coupled use of farmyard manure (FYM) and K_2_SO_4_ decreased the SAR due to the displacement of Na^+^ from the exchangeable complex [63]. Moreover, these applications also made changes in the soil bulk density, which could possibly be due to the presence of ash in the BC [63].

The current findings revealed that the addition of BC and CM significantly changed the soil organic matter, which is the most limiting factor in soils, particularly in arid areas [64]. It was observed that CM application increased the soil organic matter, although BC application reduced the soil organic matter. The increased soil organic matter with CM application could be due to the mineralization process which provides nutritive elements via manure [59] (Rahimi et al., 2023). However, BC application reduced the mineralization of SOM because of the carbon dioxide formation, which causes a decline in soil organic matter [64]. The decreasing rate of SOM mineralization may be ascribed to the extent of identical factors including aeration and nitrogen availability. This could be due to the leisurely mineralization rates in BC, which led to the immobilization of most of the nitrogen [65].

The use of various treatments of BC and CM along with K fertilizer showed a significant effect on the total nitrogen (%) in both years (Table 3). Possibly, this could be due to the effects of the manure-based biochar N mineralization rate [66]. High N contents were recorded as a result of increased net N mineralization, following the incorporation of manure-based BC [59]. These applications also showed a significant increase in soil available phosphorous. This could be attributed to the high P concentration availability in BC and CM [45]. Previously, it has been shown that BC application increased phosphorus availability in the soil [61]. In another study, it was concluded that BC application can significantly increase soil phosphorus [58]. This study also showed that BC and CM application significantly increased the soil’s available K. However, these results were more pronounced with the addition of K fertilizers. This might be due to the presence of K content in BC [62]. Application of K with organic manure increases soil K due to the conversion of exchangeable K from non-exchangeable K in soil [62]. However, some studies reported that BC application along with K had a non-significant effect on soil K concentration, which reveals that BC contains some K content [61].

## 4. Materials and Methods

### 4.1. Site Description

The site was located at latitude of 38″ E, 43°48′28.59″ N, and at an altitude of 248.5 m a.s.l in Jilin Institute of Chemical Technology, China, specifically at the College of Resource and Environment Engineering. The region has four distinct seasons and falls under a moderately semi-arid climatic zone. The average annual rainfall is 553–914 mm, with an annual temperature of 4–6 °C, and about 130–150 days without frost each year. Before sowing, the soil of the experimental site was sampled and analyzed for physio-chemical properties as presented in Table 3.

### 4.2. Experimental Design

The experiment was spread out in a randomized complete block design in replicates (i.e., 4 replications) with a split-plot arrangement. BC and CM (i.e., 2 t ha^−1^ and 4 t ha^−1^, respectively), and K fertilizer at four rates, 30, 60, 90, and 120 kg K ha^−1^, were incorporated into the soil before sowing. The BC and CM were applied in the main plots, while K was applied in the sub-plots in the form of sulphate of potash-SOP. Other fertilizers such as nitrogen (90 kg N ha^−1^) and phosphorus (120 kg P ha^−1^) were uniformly applied as basal doses to each experimental plot as urea and DAP, respectively.

**Figure 8:**
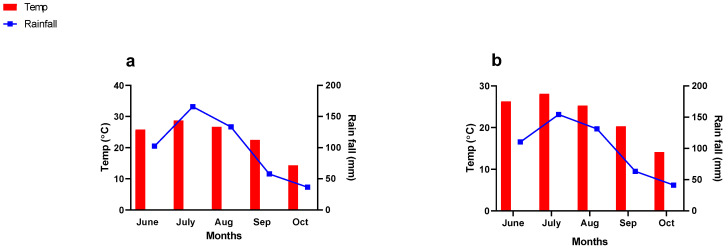
Monthly average temperature and rainfall of the experimental site during the growing season in the years (**a**) 2021 and (**b**) 2022.

Maize (Zhengdan 958) was used as a test crop because of its high-yielding potential and tolerance to drought stress. The crops were sown in early June (7 June 2021 and 11 June 2022) and harvested in late October (25 October 2021 and 29 October 2022). The plants were planted at a seed-to-seed space of 20–25 cm and row-to-row space of 60 cm. To control weeds and insect attacks, Syngenta dual gold (960 EC, herbicide) at the rate of 1.5 L ha^−1^ and FMC Furadan 3% granules (insecticide) at the rate of 20 kg ha^−1^ were applied when needed. The average monthly air temperature and rainfall were recorded throughout the growing season in 2021 and 2022 (Figure 8). Other agronomic practices such as irrigation, hoeing, weeding, etc., were performed manually when required.

### 4.3. Biochar and Cattle Manure Collection and Preparation

The BC was prepared from *Acacia nilotica* raw material, because of its high carbon content, nutrient richness, and water retention ability. The raw materials of *A. nilotica* were kept in a furnace under a pyrolysis process at 300–500 °C for 2–3 h. After pyrolysis, the BC was preserved for 24 h at room temperature for cooling to take place. Thereafter, the cooled BC was minced, using a hammer mill passed through a 2 mm sieve, and chemically analyzed in the laboratory for physio-chemical characteristics (Table 1). However, for the CM, the fresh cow dung/feces were collected from local cow/cattle farm and used as CM. The BC and CM, together with K fertilizers, were manually applied to the experimental soil before the sowing of maize.

### 4.4. Soil Sampling and Lab Analysis

The pre-sowing composite soil samples, at a soil depth of 20 cm, were collected from the experimental field to check the soil physio-chemical characteristics (Table 1). After harvesting, the soil samples (at 20 cm depth) were collected from all experimental plots, following a zigzag pattern at different positions to test the different physio-chemical characteristics of the soil. Potassium was determined according to the method of [64], whereas phosphorus was analyzed based on the method of [65]. Organic matter was examined based on the method of [66] while the soil EC, pH, and sodium adsorption ratio were analyzed based on the proposed method of [64].

### 4.5. Growth and Yield of Maize

The growth parameters, such as stem diameter (mm), plant height (cm), and number of leaves per plant, were measured at the third, fifth, and ninth leaf stages of maize. The stem diameter (mm) was measured with a Vernier caliper, plant height (cm) with a measuring tape, and the number of leaves by manually counting the leaves per plant. The number of seeds per cob, 1000-grain weight (g), and grain yield (kg ha^−1^) were measured after the plant’s harvest at full maturity, using an electronic scale.

### 4.6. Leaf Area

The leaf area of the maize crop was measured using the formula in Equation (1)

(1) $$LA({cm}^{2})=LW\times LL\times 0.7$$

where *LA* is leaf area, *LW* is leaf width, *LL* is leaf length, and 0.73 is the coefficient factor for the maize crop.

### 4.7. Chlorophyll SPAD Values and Photosynthetic Characteristics

The SPAD Chlorophyll Meter (SPAD-502, Minolta Camera, Tokyo, Japan) was employed for the assessment of chlorophyll SPAD values. On the other hand, the photosynthetic activities were measured using the Li-6400XT portable photosynthesis system (Licor Inc., Lincoln, NE, USA) [50]. The two indices (i.e., chlorophyll SPAD values and photosynthetic characteristics) were measured at the ninth leaf stage of maize crop, in the morning between 9:00 am and 11:00 am. The transpiration rate (Tr), photosynthetic activities (Pn), stomatal conductance (Gs), and intercellular carbon dioxide (CO_2_) of the maize leaves were assessed at leaf temperature of ~27 °C with the photosynthetic system adjusted at a continuous CO_2_ level of 400 μmol mol^−1^ and constant light (i.e., 80, 100, 150, 200, 400, 600, 800, and 1000 μmol m^−2^ s^−1^) [38].

### 4.8. Statistical Analysis

The collected data were computed in MS Excel and statistically analyzed using the statistical analysis software, Statistix 8.1 [66]. The data were analyzed using a split-plot experimental design by keeping the BC and CM as the main plot and K fertilizer levels as a subplot. The means were compared at the LSD (least significant difference) test at a *p* ≤ 0.05 level of probability.

## 5. Conclusions

The results of this study showed that K fertilization combined with biochar and cattle manure had improved growth and yield by modifying the soil’s physio-chemical characteristics. In general, the cattle manure integration with potassium proved more useful in terms of soil properties improvement, plant growth, and yield aspects of the crop. However, the biochar application along with an increased level of potassium fertilizer was also reasonable. This suggests that optimal potassium application, together with organic amendments such as biochar and cattle manure, could be a better option for improving not only the soil fertility but also crop growth and yield, and is crucial for viable agricultural production. Overall, integrating the use of these components fosters a sustainable agricultural production system by improving soil quality and fertility, enhancing crop yield, promoting ecological health, and reducing dependency on synthetic inputs. This integrated approach is essential for achieving long-term agricultural productivity and environmental sustainability.

## Figures and Tables

**Table 1:** Physio-chemical properties of the soil as affected by potassium (K) fertilization combined with biochar and cattle manure.

Parameter	Treatment	2021				2022			
		BC		CM		BC		CM	
**Soil pH**	Control	8.2	±0.5	8.1	±0.1	8.1	±0.1	8.5	±0.2
	30 kg K ha^−1^	7.9	±0.7	8.1	±0.2	7.4	±0.3	8.0	±0.1
	60 kg K ha^−1^	7.9	±0.1	8.0	±0.1	7.7	±0.1	8.3	±0.1
	90 kg K ha^−1^	7.8	±0.1	7.8	±0.4	7.6	±0.3	7.5	±0.3
	120 kg K ha^−1^	7.8	±0.2	8.1	±0.1	7.9	±0.2	8.3	±0.2
	LSD	0.57				0.61			
**Soil EC** **(μS cm^−1^)**	Control	660.0 a	±5.4	321.3 cde	±5.4	850.1 a	±0.8	786.4 b	±0.8
	30 kg K ha^−1^	490.1 abc	±5.0	223.0 e	±5.0	732.9 cd	±0.6	682.8 e	±0.6
	60 kg K ha^−1^	481.6 abc	±5.2	206.6 e	±5.2	672.2 e	±0.8	613.1 f	±0.8
	90 kg K ha^−1^	421.2 bcd	±1.4	344.5 bcde	±1.4	621.0 f	±0.2	639.1 f	±0.2
	120 kg K ha^−1^	508.1 ab	±4.8	244.4 de	±4.8	715.0 d	±0.6	762.0 bc	±0.6
	LSD	185.14				32.09			
**SAR**	Control	6.3 a	±0.1	5.5 a	±0.1	2.3 bcd	±0.1	2.6 bcd	±0.1
	30 kg K ha^−1^	3.9 bc	±0.5	1.5 f	±0.5	1.7 cd	±0.7	5.5 a	±0.7
	60 kg K ha^−1^	2.6 de	±0.3	4.7 b	±0.3	5.5 a	±0.5	3.1 bcd	±0.4
	90 kg K ha^−1^	3.4 cd	±0.2	2.6 de	±0.2	4.4 ab	±0.7	0.8 d	±0.7
	120 kg K ha^−1^	2.6 e	±0.3	1.4 f	±0.3	3.8 abc	±0.5	1.5 d	±0.5
	LSD	0.94				2.21			
**Bulk density** **(g cm^−3^)**	Control	1.2 ab	±0.001	1.2 ab	±0.001	1.3 ab	±0.003	1.3 ab	±0.3
	30 kg K ha^−1^	1.2 a	±0.001	1.2 ab	±0.001	1.3 ab	±0.002	1.3 b	±0.2
	60 kg K ha^−1^	1.2 a	±0.001	1.2 ab	±0.001	1.4 a	±0.005	1.2 b	±0.1
	90 kg K ha^−1^	1.2 a	±0.002	1.2 a	±0.002	1.4 ab	±0.001	1.4 a	±0.1
	120 kg K ha^−1^	1.2 b	±0.004	1.2 a	±0.004	1.2 b	±0.03	1.4 ab	±0.1
	LSD	0.01				0.15			
**Organic matter (%)**	Control	0.5 f	±0.1	0.8 c	±0.1	0.5 f	±0.1	0.8 bcd	±0.1
	30 kg K ha^−1^	0.6 d	±0.1	0.8 c	±0.1	0.7 def	±0.1	0.9 ab	±0.1
	60 kg K ha^−1^	0.5 ef	±0.1	0.9 ab	±0.1	0.7 cde	±0.1	0.8 abc	±0.1
	90 kg K ha^−1^	0.5 f	±0.1	0.9 b	±0.1	0.8 bcd	±0.1	0.9 ab	±0.1
	120 kg K ha^−1^	0.6 e	±0.1	0.9 a	±0.1	0.6 ef	±0.2	0.9 a	±0.2
	LSD	0.05				0.15			

The mean with dissimilar lowercase letters (±SD) are significantly different from each other at *p* ≤ 0.05 level of probability. K; potassium applied at different rate (i.e., 30, 60, 90 and 120 kg ha^−1^), BC; biochar, CM; cattle manure, EC; electrical conductivity, and SAR; soil absorption ratio, LSD; lease significant difference.

**Table 2:** Soil available nutrients as influenced by potassium (K) fertilization combined with biochar and cattle manure.

Parameter	Treatment	2018				2019			
		BC		CM		BC		CM	
**Total N (%)**	Control	0.02 f	±0.03	0.038 c	±0.03	0.02 f	±0.03	0.04 bcd	±0.03
	30 kg K ha^−1^	0.03 d	±0.02	0.039 c	±0.02	0.03 def	±0.02	0.04 ab	±0.02
	60 kg K ha^−1^	0.03 ef	±0.03	0.044 ab	±0.03	0.03 cde	±0.01	0.04 abc	±0.01
	90 kg K ha^−1^	0.02 f	±0.03	0.042 b	±0.03	0.04 bcd	±0.01	0.05 ab	±0.01
	120 kg K ha^−1^	0.03 e	±0.03	0.045 a	±0.03	0.03 ef	±0.04	0.05 a	±0.04
	LSD	2.6				7.6			
**Soil extractable P** **(mg kg^−1^)**	Control	6.9	±0.03	7.2	±0.03	6.2 b	±0.2	7.8 a	±0.2
	30 kg K ha^−1^	7.3	±0.1	7.8	±0.1	7.5 a	±0.01	7.6 a	±0.01
	60 kg K ha^−1^	7.4	±0.1	7.9	±0.1	7.8 a	±0.02	7.9 a	±0.02
	90 kg K ha^−1^	6.9	±0.1	7.4	±0.1	8.1 a	±0.04	7.8 a	±0.04
	120 kg K ha^−1^	7.5	±0.1	7.0	±0.1	7.2 ab	±0.03	7.4 ab	±0.03
	LSD	NS				1.3			
**Soil extractable K (mg kg^−1^) in soil**	Control	114.9 f	±1.2	154.9 de	±1.2	127.9 e	±0.2	136.0 de	±0.2
	30 kg K ha^−1^	123.3 ef	±1.6	180.0 bcd	±1.6	172.3 bcd	±0.2	182.4 bc	±0.3
	60 kg K ha^−1^	168.5 cd	±0.8	200.1 abc	±0.8	157.2 cde	±1.2	204.7 abc	±1.2
	90 kg K ha^−1^	189.0 abc	±0.5	209.1 a	±0.5	196.8 abc	±0.6	221.3 a	±0.6
	120 kg K ha^−1^	200.0 abc	±0.1	203.4 ab	±0.1	212.9 ab	±0.1	213.0 ab	±0.004
	LSD	33.1				48.8			

The mean with dissimilar lowercase letters (±SD) are significantly different from each other at *p* ≤ 0.05 level of probability. N; nitrogen, P; phosphorous, K; potassium applied at different rate (i.e., 30, 60, 90 and 120 kg ha^−1^), BC; biochar, CM; cattle manure, LSD; least significant difference, NS; non-significant.

**Table 3:** Physical and chemical properties of soil, water, biochar, and cattle manure before sowing.

Soil Parameter	Soil	BC	CM
pH	7.5	8.2	7.8
Electrical conductivity (μs cm^−1^)	7.3	2490	80.1
Bulk density (g cm^−3^)	1.3	0.5	0.3
Sodium adsorption ratio (SAR)	5.1	4.3	0.5
Potassium (mg kg^−1^)	626.8	105.2	30.4
Sodium (mg kg^−1^)	0.4	530	123.6
Phosphorus (mg kg^−1^)	7.0	14.7	31.8

BC; biochar, and CM; cattle manure.

## Data Availability

The data can be available on request to the corresponding author.
